# HLA-G and CD152 Expression Levels Encourage the Use of Umbilical Cord Tissue-Derived Mesenchymal Stromal Cells as an Alternative for Immunosuppressive Therapy

**DOI:** 10.3390/cells11081339

**Published:** 2022-04-14

**Authors:** Bernardo Zoehler, Letícia Fracaro, Lidiane Maria Boldrini-Leite, José Samuel da Silva, Paul J. Travers, Paulo Roberto Slud Brofman, Maria da Graça Bicalho, Alexandra Cristina Senegaglia

**Affiliations:** 1 Immunogenetics and Histocompatibility Laboratory, Department of Genetics, Universidade Federal do Paraná (UFPR), Curitiba 81530-001, PR, Brazil; samuel-bio@hotmail.com (J.S.d.S.); mgbicalho@gmail.com (M.d.G.B.); 2 Core for Cell Technology, School of Medicine, Pontifícia Universidade Católica do Paraná (PUCPR), Curitiba 80910-215, PR, Brazil; leticiafracaro@gmail.com (L.F.); lidiane.leite@pucpr.br (L.M.B.-L.); paulo.brofman@pucpr.br (P.R.S.B.); 3 National Institute of Science and Technology for Regenerative Medicine, INCT-REGENERA, Rio de Janeiro 21941-902, RJ, Brazil; 4 Centre for Regenerative Medicine, Institute for Regeneration and Repair, The University of Edinburgh, Edinburgh EH16 4UU, UK; paul.travers@ed.ac.uk

**Keywords:** mesenchymal stromal cell, immunosuppressive therapy, HLA-G molecule, umbilical cord tissue, adipose tissue, dental pulp tissue, costimulatory molecules, immunosuppressive molecules

## Abstract

Mesenchymal stromal cells (MSCs) have been used in immunosuppressive therapy due to their therapeutic effects, with the HLA-G molecule seeming to play a fundamental role. This work evaluated alternative MSC sources to bone marrow (BM), namely, umbilical cord tissue (UC), adipose tissue (AD) and dental pulp tissue (DP), and the influence of interferon-γ (IFN-γ) and hypoxia on the cultivation of these cells for use in immunosuppression therapies. Expression of costimulatory markers CD40, CD80 and CD86 and immunosuppressive molecules CD152 and HLA-G was analyzed. Lymphocyte inhibition assays were also performed. Sequencing of the HLA-G gene from exons 1 to 5 was performed using next-generation sequencing to determine the presence of alleles. UC-derived MSCs (UCMSCs) expressed higher CD152 and HLA-G1 under standard cultivation. UCMSCs and DP-derived MSCs (DPSCs) secreted similar levels of HLA-G5. All MSC sources inhibited the proliferation of peripheral blood mononuclear cells (PBMCs); growth under regular versus hypoxic conditions resulted in similar levels of inhibition. When IFN-γ was added, PBMC growth was inhibited to a lesser extent by UCMSCs. The HLA-G*01:04:01:01 allele appears to generate a more efficient MSC response in inhibiting lymphocyte proliferation. However, the strength of this conclusion was limited by the small sample size. UCMSCs are an excellent alternative to BM in immunosuppressive therapy: they express high concentrations of inhibitory molecules and can be cultivated without stimuli, which minimizes cost.

## 1. Introduction

Autoimmune diseases affect approximately 2–5% of the world’s population and are of great medical and economic importance. These disorders are mainly mediated by antibodies and are usually chronic, which can be debilitating. In short, an autoimmune disease occurs when the individual’s immune system starts to produce antibodies that target its own molecules [1]. Proinflammatory cytokines secreted by M1 macrophages, Th1 cells and Th17 cells play a fundamental role in initiating autoimmune diseases. In contrast, anti-inflammatory cytokines decrease inflammation, relieving symptoms [1,2].

Although conventional therapy has evolved, with biological agents being more selective for cells of the immune system, immunotherapeutic regimes that target common pathways of the immune system inevitably cause undesirable consequences [3], leading to serious side effects, and these approaches are often inefficient in treating the disease [1]. Thus, alternative therapies are needed. Infusion of stromal cells has recently been used to treat autoimmune diseases, including systemic lupus erythematosus, Sjögren’s syndrome, scleroderma and rheumatoid arthritis, demonstrating safety and efficiency [4]. Hence, immunosuppressive therapy with MSCs is being strongly considered in clinical trials as a possible treatment for autoimmune diseases.

In contrast to the initial idea that MSCs would fulfil their therapeutic potential through their differentiation capacity for cellular tissue replacement, the desired therapeutic effects are being triggered by secretion of extracellular molecules and vesicles, locally or at a distance [5]. Among the expressed proteins, human leukocyte antigen-G (HLA-G) appears to play a central role in modulating the immune response, with other cytokines playing auxiliary functions [6,7].

Unlike other proteins in the HLA complex, the primary role of HLA-G is not antigen presentation. HLA-G has an immunosuppressive role, inhibiting cytotoxic activity and inducing proliferation of suppressor cells [8,9]. HLA-G has the following functions: (i) inhibiting the lytic activity and proliferation of natural killer (NK) cells through its interaction with killer cell immunoglobulin-like receptors 2DL4 (KIR2DL4) and Ig-Like Transcript 2 (ILT2); (ii) inhibiting proliferation of T and B cells through ILT2 receptors; (iii) stimulating differentiation of T and B cells into suppressor cells (also known as regulatory T and B cells, respectively) through ILT2 receptors; and (iv) inhibiting maturation of dendritic cells (DCs) and antigen-presenting cells (APCs) by binding to Ig-like transcript 4 (ILT4) and ILT2 receptor [10,11,12,13].

CD152 (also known as cytotoxic T lymphocyte antigen 4—CTLA-4), which downregulates T-cell activation [14], is another critical molecule for maintaining the immune response mediated by MSCs. CD152 has a greater affinity for CD80 (B7.1) and CD86 (B7.2) present on DCs, competing with CD28 molecules on T cells for binding to these ligands and thus preventing T-cell co-stimulation [15,16]. CD152 and HLA-G effectively block proinflammatory factor secretion by peripheral blood mononuclear cells (PBMCs), playing an essential role in the immunosuppressive effect mediated by MSCs [16,17]. They also guarantee the T-cell anergic state, induce apoptosis and inhibit proliferation of immune cells [15].

Overall, the expression of cytokines by pericytes (MSCs in vivo) is influenced by the environment in which the cells are found in vivo. In vitro cultivation under different conditions can trigger differential expression of these factors [18,19]. Stress caused by decreased oxygen (hypoxia) results in activation of hypoxia-inducible factor 1-alpha (HIF-1), which induces transcription of specific genes. Cultivation under hypoxic conditions induces the expression of growth factors such as vascular endothelial growth factor (VEGF), fibroblast growth factor 2 (FGF-2), hepatocyte growth factor (HGF) and insulin-like growth factor 1 (IGF-1) by MSCs from the bone marrow (BM), adipose tissue (AD), placenta and DP. Regarding their immunosuppressive characteristics, hypoxia can increase expression in MSCs of indoleamine 2,3-dioxygenase (IDO), an essential molecule for modulation of the immune response [19,20].

In addition to hypoxia, secretion of immunosuppressive factors is influenced by an inflammatory environment. IFN-γ is a proinflammatory cytokine produced by immune cells and is associated with the development of acute graft versus host disease (GVHD). Simulation of an inflammatory environment by the addition of IFN-γ can induce expression of IDO, transforming growth factor-beta (TGF-β), HGF and prostaglandin E2 (PGE2) [19,21,22]. Thus, it is plausible that the conditioning of MSCs under hypoxia or IFN-γ stimulation increases their immunosuppressive properties and can benefit allotransplantation or immune disease treatment.

Due to the immunomodulatory potential of MSCs, in addition to the lack of expression of the stimulatory molecules CD40, CD80, CD86 and HLA-DR by these cells [6,23], MSCs have become agents of immunosuppressive therapy for immunological diseases. Infusion of MSCs has been tested as an alternative form of immunosuppressive treatment in several immune disorders, such as GVHD, type 1 diabetes, Crohn’s disease and multiple sclerosis [24].

Thus, identifying a source of MSCs that exhibits higher expression of inhibitory molecules (HLA-G and CD152), lower expression of costimulatory molecules (CD40, CD80 and CD86) and consequently a more significant potential to inhibit proliferation of T lymphocytes may be of value for the treatment of diseases that require immune response modulation. Similarly, determining culture conditions that stimulate the expression of these inhibitory molecules may allow MSCs to be grown under conditions that amplify their immunosuppressive potential and thus enhance their therapeutic application.

## 2. Materials and Methods

### 2.1. Cell Culture and Expansion

MSCs were isolated from 9 healthy individuals: three samples were from umbilical cord (*n* = 3) (age = newborn), called umbilical cord mesenchymal stromal cells (UCMSCs); three from adipose tissue (*n* = 3) (mean age = 48.33 ± 21.51), called adipose tissue-derived stromal cells (ADSCs); and three from dental pulp (*n* = 3) (mean age = 24.67 ± 4.51), called dental pulp stromal cells (DPSCs). Informed consent from the volunteers was obtained following the code of ethics in research adopted by the Research Ethics Committee of Pontifícia Universidade Católica do Paraná (CAAE 98834718.9.0000.0020).

The protocols used in previous studies by our group were followed to collect and isolate dental pulp, adipose tissue [25] and umbilical cord [26] cells. The cells were cultured at a density of 1 × 10^5^ MSCs/cm^2^ in 75-cm^2^ culture flasks (TPP, Trasadingen, Switzerland) and incubated in a humidified incubator at 37 °C with 5% CO_2_. UCMSCs and DPSCs were grown in IMDM (Gibco^TM^, Invitrogen, New York, NY, USA) supplemented with 20% FBS (Gibco^TM^, Invitrogen, New York, NY, USA), 100 μg/mL streptomycin (Gibco^TM^, Invitrogen, NY, USA) and 100 U/mL penicillin (Gibco^TM^, Invitrogen, New York, NY, USA). ADSCs were grown at the same density with DMEM/F-12 medium (Gibco^TM^, Invitrogen, New York, NY, USA) supplemented with 20% FBS, 100 μg/mL streptomycin and 100 U/mL penicillin. The culture medium was changed every 3–4 days until the cells reached confluence, after which they were dissociated with trypsin/0.25% EDTA (Invitrogen^TM^, New York, NY, USA) and recultured at a concentration of 1 × 10^5^ MSCs/cm^2^. Except for the population doubling (PD) calculation, cells in passages between P5 and P7 were used.

MSC proliferation rates were compared, and the following formula was used to calculate the population doubling (PD) of P4 to P5 cells:

$$\mathrm{PD}=\frac{log\left(Cf\right)-log\left(Ci\right)}{log\left(2\right)}$$

where *Cf* = final cell concentration; and *Ci* = initial cell concentration.

After reaching the required number of cells, the MSCs were separated into three groups: (1) control, maintained in standard culture (IMDM + 20% FBS); (2) IFN-γ, in which the cells were cultured with IFN-γ (10 ng/mL) for 48 h; and (3) hypoxia, with 5% CO_2_, 2% O_2_ and 93% N_2_ for 24 h.

### 2.2. Cell Characterization

The parameters recommended by the International Society for Cell and Gene Therapy (ISCT) were followed for cell characterization, which include verification of the following minimum criteria: (i) adherence of MSCs to plastic surfaces when maintained in culture; (ii) positive expression (≥95%) of CD105, CD73 and CD90 markers; and (iii) absence of expression (≤2%) of CD45, CD34, CD14, CD19 and HLA-DR cell surface markers. In addition to the markers required by ISCT, the expression of CD29 and CD166, suggested as being positive in MSCs, was analyzed [27,28,29]. We also assessed the costimulatory molecules CD40, CD80 and CD86 to evaluate the ability of MSCs to stimulate T-cell proliferation.

A million cells were transferred to each tube and washed with PBS. After washing, the supernatant was discarded by manual inversion, and corresponding antibodies (all from BD Pharmingen) against the following were used according to the manufacturer’s instructions: FITC-labelled CD14 (BD#555397), CD45 (BD#555482), CD19 (BD#555412), CD73 (BD#550257), CD90 (BD#555596), CD166 (BD#559263) and CD40 (BD# 555588), PerCP-labelled HLA-DR (BD#551375), Pe-Cy5-labelled CD80 (BD#559370) and APC-labelled CD34 (BD#555824), CD105 (BD#562408), CD29 (BD#559883) and CD86 (BD#555660). After a 30 min incubation period, the cells were again washed with PBS and fixed with 1% paraformaldehyde (PFA). At least 100,000 events were acquired using a BD FACSCalibur^TM^ (BD Biosciences, San Jose, CA, USA), and the analysis was performed using FlowJo software (FlowJo, Ashland, TN, USA).

### 2.3. Labelling of CD152

For CD152 labelling (BD Pharmingen^TM^, BD Biosciences, San Jose, CA, USA), 1 × 10^6^ cells were washed with 1 mL PBS and incubated with Fix & Perm Medium A solution (Invitrogen^TM^, Carlsbad, CA, USA) for 15 min at room temperature in the dark for permeabilization of the cell membrane. The cells were then rewashed with PBS and incubated with Fix & Perm Medium B and CD152 (PE-Cy5) antibody for 30 min at room temperature in the dark. Afterwards, the cells were washed with PBS and fixed with 1% paraformaldehyde (PFA). Cell acquisition was performed using a FACS Calibur cytometer (BD Biosciences, San Jose, CA, USA), and the analysis was performed using FlowJo software (FlowJo, Ashland, TN, USA).

### 2.4. Labelling of HLA-G1

For HLA-G1 labelling, 1 × 10^6^ cells were washed with 1 mL PBS. The supernatant was discarded by manual inversion, and an anti-MEM-G/9 (PE) antibody (EXBIO^TM^, Praha, Czech Republic) was added and incubated for 30 min in the dark at room temperature. The cells were next rewashed with PBS and fixed with 1% PFA. Cell acquisition was performed using a FACS Calibur cytometer (BD Biosciences, San Jose, CA, USA), and the analysis was performed using FlowJo software (FlowJo, Ashland, TN, USA).

### 2.5. HLA-G5 Detection

Soluble HLA-G in the supernatant of MSC cultures was measured by ELISA (enzyme-linked immunosorbent assay). Each well of a 96-well plate was coated with 100 µL of diluted MEM-G/9 capture antibody and incubated for 16 h. After washing, 200 µL of blocking buffer (Diluent DAKO) was added and incubated for 1 h. After washing, 100 µL of the sample was incubated for two hours. After washing, 100 µL of anti-human β2-microglobulin antibody tagged with horseradish peroxidase (HRP) was added and incubated for 60 min. After another wash, 100 µL of TMB substrate was added to react with the HRP conjugate. The reaction was stopped by acidic solution addition (100 µL of HCl 1N), followed by absorbance measurement at 450 nm (proportional to the concentration of sHLA-G). Analysis of the HLA-G concentration was performed using GraphPad Prism 7^®^ software.

### 2.6. Lymphocyte Inhibition Assay

Lymphocyte Inhibition Assay was performed to compare the immunosuppressive potential between MSC sources. The assay analyzes the rate of PBMCs labelled with carboxyfluorescein succinimidyl ester (CFSE) (Sigma–Aldrich^TM^, St. Louis, MO, USA), stimulated with 1.0 µg/mL of the mitogen phytohemagglutinin (PHA) (Sigma–Aldrich^TM^, St. Louis, MO, USA) and cultivated in the presence of MSCs to evaluate the inhibition potential of these cells.

For each experiment, a control plate was prepared in which PBMCs were grown in triplicate as follows: (i) PBMCs not labelled with CFSE and not stimulated with PHA; (ii) CFSE-labelled and non-PHA-stimulated PBMCs; and (iii) CFSE-labelled and PHA-stimulated PBMCs. To assess the immunosuppressive potential of MSCs, the cells were cultured in 24-well plates and incubated for two hours for adherence. PBMCs obtained from a healthy volunteer donor were isolated and incubated with CFSE for cell labelling. PBMCs (1 mL) were added to each well containing MSCs, followed by stimulation with PHA. PBMCs were added at different concentrations (MSCs:PBMCs = 1:2; 1:10). The plates were incubated for five days at 37 °C protected from light. After five days, the PBMCs were collected and incubated with CD3 (for lymphocyte labelling) (BD Pharmingen^TM^, BD Biosciences, San Jose, USA) for 15 min, and cell acquisition was performed using a FACS Calibur cytometer (BD Biosciences, San Jose, CA, USA). The analysis was performed using FlowJo software (FlowJo, Ashland, TN, USA).

The following formula was used for the calculation of the inhibition rate:

$$\%inhibition=100-\left(\frac{\overset{`}{x}1x100}{\overset{`}{x}2}\right)$$

where$\;\overset{`}{x}1$ = average marking of PBMCs co-cultivated with MSCs; and $\overset{`}{x}2\;$= average score for the PBMC control.

### 2.7. HLA-G Genotyping

The commercial kit SBTexcellerator^®^ HLA-G (GenDx^TM^, Utrecht, The Netherlands) was used to amplify the coding region, and the manufacturer’s recommendations were followed. The coverage region for sequencing the HLA-G gene included the coding sequences from exons 1 to 5, which enabled the determination of allelic variants and genotypes. Sequencing was performed by next-generation sequencing (NGS) through the MiniSeq System platform (Illumina^TM^, San Diego, CA, USA). To align reads, Twin software from Omixon, Inc. (Budapest, Hungary) was used based on two algorithms for sequence alignment compared with the ImMunoGeneTics (IMGT) database for allele definition.

### 2.8. Statistical Analysis

The Kruskal–Wallis test with Dunn’s multiple comparisons test was performed to compare expressions. The analyses were performed with GraphPad Prism version 8.4.0 software. Graphs were made using Python’s Seaborn (version 0.11.2) library (version 3.7.7) and GraphPad Prism (version 8.4.0).

## 3. Results

### 3.1. Cell Characterization

**Figure 1:**
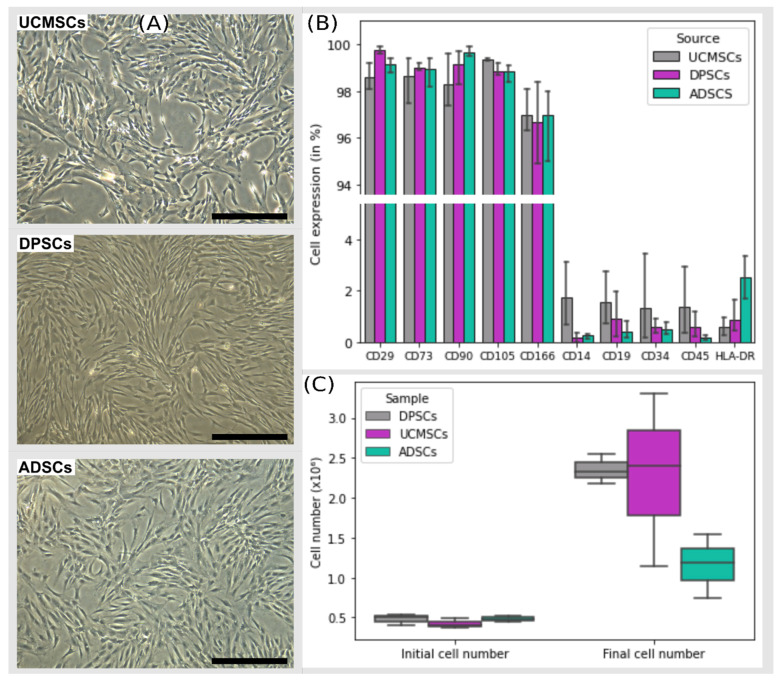
Representative image of MSC cultures from different sources and cell characterization. Legend: (**A**) Cultivation of MSCs from umbilical cord (UCMSCs), dental pulp (DPSCs) and adipose tissue (ADSCs). Magnification: 5×; black bar: 500 µm. (**B**) Positive and negative expression profiles of membrane markers required by the International Society for Cell and Gene Therapy for three different sources of MSCs. (**C**) Population doubling (PD) of UCMSCs, DPSCs and ADSCs from P4 to P5. No significant difference in PD was observed between sources (*p* = 0.193).

MSCs showed fibroblastoid morphology and adherence to plastic (Figure 1A). Immunophenotypic characterization of cell surface antigen markers on the MSCs indicated the same pattern for the various sources. More than 95% of the cells were positive for CD105, CD73, CD29, CD166 and CD90, and less than 2% expressed CD14, CD19, CD34, CD45 and HLA-DR (Figure 1B). PD was calculated for cells at P4 to P5. UCMSCs had the highest value for population doubling (PD = 1.5986) and ADSCs had the lowest value (PD = 1.1106). Nevertheless, there was no significant difference between the sources (*p* = 0.193) (Figure 1C).

**Figure 2:**
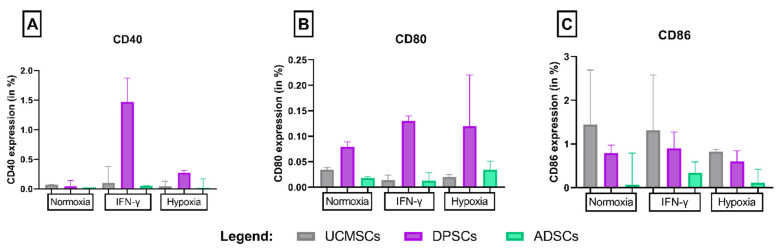
Expression of costimulatory molecules CD40, CD80 and CD86. Legend: Expression of (**A**) CD40, (**B**) CD80 and (**C**) CD86 on umbilical cord (UCMSCs), dental pulp (DPSCs) and adipose tissue (ADSCs).

CD40, CD80 or CD86 costimulatory molecules, which are implicated in immune system activation, were not expressed before or after activation. Variations between samples, different sources and treatments were not significantly different (*p* < 0.05). MSCs expressed low levels (less than 2%) of the costimulatory markers CD40, CD80 and CD86, regardless of the source from which the cells were obtained (Figure 2).

### 3.2. CD152

**Figure 3:**
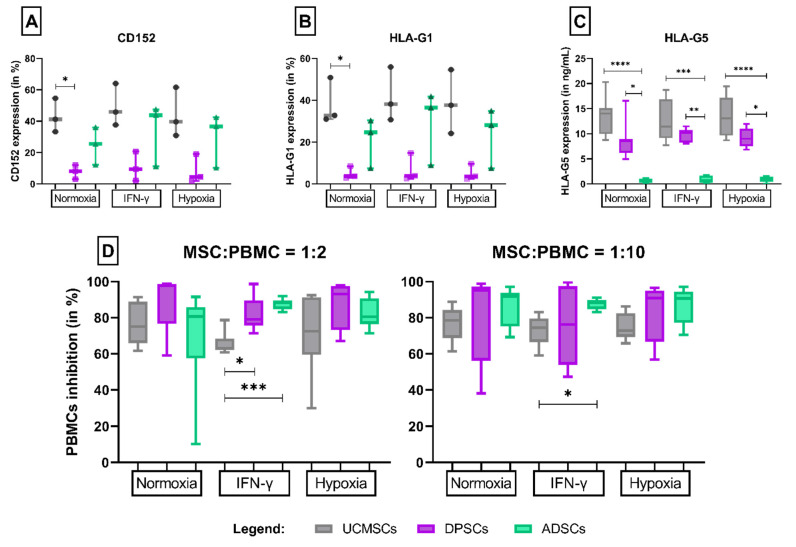
Inhibitory profile of MSCs cultured in standard medium, stimulated with IFN-γ, or under hypoxic conditions. Legend: Differential expression of (**A**) CD152, (**B**) HLA-G1 membrane isoform and (**C**) HLA-G5 soluble isoform between sources of MSCs cultured in different culture conditions; (**D**) ability to inhibit the proliferation of immune cells by MSCs in different culture conditions with the proportion MSCs:PBMCs 1:2 and 1:10. *p* values: (*) ≤ 0.05; (**) ≤ 0.01; (***) ≤ 0.001; (****) ≤ 0.0001. UCMSCs = umbilical cord mesenchymal stromal cells; DPSCs = dental pulp stromal cells; ADSCs = adipose tissue-derived stromal cells; INF-y = interferon-gamma; MSCs = mesenchymal stromal cells; PBMCs = peripheral blood mononuclear cells.

Cultivated under standard conditions, UCMSCs expressed the highest levels of CD152. Expression of this inhibitory molecule by UCMSCs (41.2%) was significantly higher than that by DPSCs (8.19%, *p* = 0.0338). DPSCs expressed the lowest levels of CD152 in culture with IMDM (8.19%), with IFN-γ (9.52%) and hypoxia (4.57%). When cultivated under IFN-γ stimulation or with a low O_2_ concentration, all MSC sources expressed similar levels of this molecule. The grouping of data according to the culture method allowed us to observe that the presence of IFN-γ or hypoxia did not induce an increase in CD152 expression by UCMSCs (*p* = 0.8286), DPSCs (*p* = 0.8786) or ADSCs (*p* = 0.6286) (Figure 3A).

### 3.3. HLA-G: -G1 and -G5 Isoforms

Analysis of HLA-G1 expression revealed the highest levels of HLA-G1 expression by UCMSCs and ADSCs. The DPSC source resulted in the lowest expression of HLA-G, significantly lower than UCMSC expression in the control culture (Figure 3B). Concerning the -G5 isoform, ADSCs expressed the lowest levels, regardless of the culture method. Under standard culture conditions, ADSCs secreted significantly less HLA-G than UCMSCs (*p* < 0.0001) and DPSCs (*p* = 0.0355). After IFN-γ stimulation, ADSCs still expressed HLA-G5 at significantly lower levels than UCMSCs (*p* = 0.0003) and DPSCs (*p* = 0.0065). The same was observed in cells grown under low O_2_ concentrations. In general, culture conditions did not influence the expression of HLA-G1 or HLA-G5 (Figure 3C).

### 3.4. Lymphocyte Inhibition Assay

Independent of culture conditions, all MSCs inhibited proliferation of PBMCs at levels adequate for clinical use (inhibition ≥ 50%). The MSCs obtained from all sources similarly inhibited PBMCs when grown under standard cultivation conditions or at low O_2_. However, when IFN-γ was added, the growth of PBMCs was significantly less inhibited by the presence of UCMSCs than DPSCs (1:2, *p* = 0.0251) and ADSCs (1:2, *p* = 0.0002; 1:10, *p* = 0.0157) (Figure 3D).

### 3.5. HLA-G Expression Associated with Genotype

**Figure 4:**
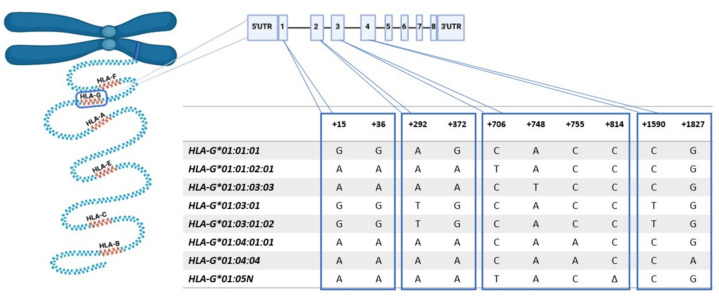
HLA-G genotyping: Nucleotide sequences of the eight alleles found in this study (IMGT version 3.46.0, 11 October 2021).

Eight alleles were found among the samples: HLA-G*01:01:01, G*01:01:02:01, G*01:01:03:03, G*01:03:01, G*01:03:01:02, G*01:04:01:01, G*01:04:04 and G*01:05N. The alleles were grouped into six related groups for association analysis between HLA-G genotype and phenotype: G*01:01:01G, G*01:01:02G, G*01:01:03G, G*01:03:01G and G*01:04:01G, plus the null HLA-G*01:05N allele. Identical nucleotide sequences define groups, and the exons encode the peptide-binding domain (Figure 4). No associations were observed between HLA-G genotype and protein expression. The HLA-G1 isoform was similarly expressed regardless of genotype (*p* = 0.2262). The same was observed for the soluble isoform HLA-G5 (*p* = 0.4048). Subsequently, we analyzed the association between genotype and the capacity to inhibit lymphocytes, and all genotypes inhibited mononuclear cells at similar levels (*p* = 0.1281).

## 4. Discussion

Immune cells, mainly APCs, express the molecules CD40, CD80 and CD86. Together with MHC, these molecules activate the immune system [23]. They have an affinity for CD28 receptors and when expressed by cells, can trigger a costimulatory activation signal for T cells. On the other hand, the absence of these markers does not induce activation of immune cells, maintaining them in their anergic state [30]. In an inflammatory context, MSCs can behave similar to antigen-presenting cells. For this, cells need to capture and process the antigen in addition to HLA class II expression. However, even after activation with proinflammatory factors, MSCs capable of capturing and processing antigens do not induce proliferation of specific effector T-cell clones [31]. Furthermore, in the present study, expression of CD40, CD80 and CD86 was absent or low regardless of the source and cultivation conditions, and these cells can thus be used in cell therapy [30,32,33,34].

In addition to the immune response associated with costimulatory molecules, inhibitory molecules play a crucial role in maintaining immune balance. Little is known about the involvement of CD152 in the immunomodulatory potential of MSCs. Nevertheless, this molecule is associated with IDO expression by APCs and the maintenance of an immunosuppressed environment [35]. CD152+ MSCs can strongly inhibit T-cell proliferation and prevent secretion of IL-2 and IFN-γ by immune cells [15]. In the present study, UCMSCs expressed the largest levels of CD152 under standard culture conditions. Neither UCMSCs, ADSCSs nor DPSCs were influenced by IFN-γ stimulation or hypoxia. In the study by Gaber et al. [16], low concentrations of O_2_ increased expression of CD152 and modified the splicing profile of the protein, leading to greater expression of the soluble isoform by bone-marrow-derived MSCs.

CTLA-4 can be expressed as four splicing variants, full-length (flCTLA-4), ligand-independent (liCTLA-4), soluble (sCTLA-4) and 1/4 CTLA-4 (which lacks both the ligand-binding domain and transmembrane domain), with the sCTLA-4 isoform being the predominant form expressed in MSCs. Furthermore, the amount of oxygen present during the cultivation of MSCs affects expression of the protein [16]. However, this pattern may not have been observed in the present study because the protein was evaluated as its intracellular form. Gaber et al. observed that hypoxic conditions (for 72 h) induce expression of the soluble isoform sCTLA-4 but not flCTLA-4.

Similar to T cells, CTLA-4 suppresses the NK-cell response [36]. It has been observed that tumor cells can upregulate inhibitory receptors to inhibit NK-cell responses. Treg cells are recruited into the tumor environment and inhibit NK-cell responses through CTLA-4 [37]. CTLA-4 is expressed in activated mouse NK cells, and CLTA-4 expression inhibits IFN-γ production by NK cells [38]. Little is known about the involvement of CD152 in the immunosuppressive potential of MSCs.

Furthermore, HLA-G has been shown to play a crucial role in the immunosuppressive potential exerted by MSCs, given the reversal of the inhibitory state after blocking these molecules [39,40]. As observed in the present study, UCMSCs and ADSCs expressed the highest values of HLA-G1, rendering them strong candidates for clinical application. These results align with expectations, as HLA-G is expressed constitutively in MSCs from different sources, such as Wharton’s jelly, bone marrow, menstrual blood, adipose tissue, the fetal liver, the placenta and periodontal ligaments [41]. Moreover, addition of IFN-γ did not influence expression of HLA-G by UCMSCs, DPSCs and ADSCs. Other studies reported similar results regarding membrane HLA-G expression, even when using 2.5 times higher concentrations of the proinflammatory cytokine, though soluble HLA-G was stimulated by that treatment [23,42].

Although some studies have shown upregulation of HLA-G after stimulation with IFN-y [39], there is still no consensus on the regulatory elements upstream of the translational start site of this gene. Specifically, the sequence of the IFN-stimulated response element (ISRE) of HLA-G shows great divergence from the ISRE consensus sequence of HLA class I genes. Thus, binding of interferon regulatory factors may be impeded, resulting in lack of functionality of the HLA-G gene ISRE region [43]. HLA-G primarily prevents NK-cell cytotoxicity and T-cell cytotoxicity and induces tolerance. HLA-G interacts with KIR2DL4, ILT2 and ILT4 receptors, which are expressed by NK cells, T cells and macrophages. Interaction of cells expressing HLA-G with NKs has been shown to inhibit cytotoxicity and induce production of cytokines, such as IL-6 and IL-8 [44,45,46].

Regarding soluble expression of HLA-G (isoform –G5), higher expression was observed in UCMSCs and DPSCs, though ADSCs secreted smaller amounts of HLA-G. Overall, cultivation conditions did not influence the expression of this isoform. The results obtained agree with those observed by Kim et al. [6], with no difference in expression observed for MSCs from different sources stimulated with IFN-γ. However, in that study, differential analysis of HLA-G isoforms was performed after stimulation. It was observed that in the inactive state, all MSCs expressed HLA-G1 and G7; when stimulated, (i) MSCs of the periodontal ligament expressed HLA-G1, G5 and G7; (ii) MSCs from umbilical cord expressed HLA-G5, G6 and G7; and (iii) MSCs from adipose tissue expressed HLA-G5 and G7. Such analysis allows us to infer the plasticity of MSCs responding to the microenvironment [6,47].

During cultivation at low O_2_, the stress caused by insufficient oxygen induces the formation of the HIF-1 complex that binds to hypoxia-responsive elements (HREs) within specific promoter regions, including HLA-G, stimulating transcription. Therefore, induction of HLA-G expression was expected [48]. However, hypoxia maintained HLA-G levels similar to those of other growth conditions. Nonetheless, the observation of similar values of HLA-G expression in MSCs grown at low O_2_ in the current study agrees with the work of Wobma et al. [49], who reported similar expression of HLA-G between MSCs grown in standard cultivation and under hypoxia. Thus, normoxic conditions can be considered ideal for obtaining good therapeutic results in the clinical scenario, even though preconditioning of MSCs seems to induce expression of other immunosuppressive proteins, such as IDO and PD-L1 [49,50].

The immunomodulatory potential of MSCs is not the result of a single molecule but rather the synergistic action of several factors [49,50,51,52,53]. In vitro results show the effectiveness of MSCs in inhibiting proliferation of T, B and NK cells [39,54], inducing proliferation of regulatory T cells [9], and preventing DC maturation and activation of NK cells [53]. We found no difference in the inhibition of lymphocytes by MSCs from different sources, except for cells cultured with IFN-γ. Under these conditions, DPSCs and ADSCs inhibited immune cells to a greater extent than UCMSCs, which resulted from stimulation of expression of other inhibitory molecules.

Enhancing the immune effect, pretreatment of MSCs with inflammatory cytokines is often used to increase the immunomodulatory activity of mesenchymal stromal cells. This activates the expression of IDO and other immunomodulatory factors before injection. IFN-γ is one of the most commonly used cytokines for MSC stimulation before cell transplantation. Unlike what was observed for UCMSCs in our study, pretreatment with IFN-γ positively regulates expression of immunosuppressive factors [55]. In addition, it has been shown that the effects of pretreatment of UCMSCs with IFN-γ are transient and result in a decreased ability to suppress T-cell proliferation over time [56], which may explain our results.

Finally, genotyping analyses were performed and related to lymphocyte inhibition. Although no difference in HLA-G expression according to the sample genotype was observed, there was a tendency towards increased inhibition by the G*01:04:01 allele. In the present study, the genotype G*01:01:03/G*01:04:01 showed low HLA-G expression, perhaps due to the dominant nature of the G*01:01:03 allele over the G*01:04:01 allele [57]. However, in the absence of the dominant G*01:01:03 allele, the G*01:04:01 allele is associated with high HLA-G expression [58], which might explain the increased inhibition in our assays. The same was observed in the presence of the null allele G*01:05N. The small sample size, however, only allows us to hypothesize. Therefore, we encourage the development of new studies, with a more significant number of samples dedicated to studying the association between the HLA-G genotype and gene expression, especially for the G*01:04:01 allele.

Based on the results obtained, the HLA-G*01:04:01:01 allele appears to be associated with a more efficient MSC inhibition of lymphocyte proliferation. Although this study was of an exploratory nature, which justifies the sample size [39,50,59,60,61], the strength of this conclusion is limited by the small sample size. Indeed, the small number of samples must be increased in future studies, allowing greater statistical power in comparisons. Further studies of the immunosuppressive potential of HLA-G molecules in relation to the HLA-G genotype of the donor can not only help in choosing a better source for obtaining cells but also in determining the best donor.

Another limitation of our study is related to the age difference of the donors. Previous studies have shown that the age of MSCs donors can be a critical factor affecting cell therapy outcomes and protein expression [62,63]. However, the choice of donors with the same age for the different sources studied is quite difficult. Furthermore, third molar surgery is normally performed (at least for our donors) during adolescence and early adulthood, and adipose tissue samples are more often obtained from adults who undergo the liposuction process. Once again, age differences may be another point for choosing UCMSCs as a good source of use in cell therapy because they are young cells with little or no influence from the environment.

## 5. Conclusions

Obtaining bone marrow cells involves stricter ethical issues, as it is an invasive surgical procedure. Bone marrow is the gold standard when referring to clinical trials for the treatment of autoimmune diseases, mainly graft-versus-host disease (GVHD). Our objective with this work was to identify alternative sources that are easier to obtain and that could be readily available in cases of need.

Given the results and focusing on clinical use in situations requiring regulation of the immune response, we infer positive results for DPSCs and ADSCs. Nevertheless, future work should be conducted to evaluate the therapeutic potential of UCMSCs compared to that of MSCs from bone marrow. UCMSCs proved to be an excellent alternative to bone-marrow-derived MSCs. UCMSCs express higher levels of inhibitory molecules and can be cultivated without stimuli (which minimizes the cost of cultivation). In addition, UCMSCs are obtained from material discarded after childbirth, making this source easier to obtain with no ethical consideration regarding its use.

## Data Availability

Not applicable.
